# Fn14 overcomes cisplatin resistance of high-grade serous ovarian cancer by promoting Mdm2-mediated p53-R248Q ubiquitination and degradation

**DOI:** 10.1186/s13046-019-1171-6

**Published:** 2019-04-25

**Authors:** An-Yue Wu, Li-Ying Gu, Wei Cang, Meng-Xing Cheng, Wen-Jing Wang, Wen Di, Lei Huang, Li-Hua Qiu

**Affiliations:** 1 Department of Obstetrics and Gynecology, Ren Ji Hospital, School of Medicine, Shanghai JiaoTong University, Shanghai, 200127 China; 2 Shanghai Key Laboratory of Gynecologic Oncology, Shanghai, 200127 China; 3 State Key Laboratory of Oncogenes and Related Genes, Shanghai Cancer Institute, Ren Ji Hospital, School of Medicine, Shanghai Jiao Tong University, Shanghai, 200127 China; 4 Department of Histoembryology, Genetics and Developmental Biology, Key Laboratory of Cell Differentiation and Apoptosis of Chinese Ministry of Education, Shanghai Key Laboratory of Reproductive Medicine, Shanghai Jiao Tong University School of Medicine, 280 South Chongqing Road, Shanghai, 200025 China

**Keywords:** HGSOC, Cisplatin resistance, Fn14, p53-R248Q, Hsp90

## Abstract

**Background:**

High-grade serous ovarian cancer (HGSOC) is the most lethal of all gynecological malignancies. Patients often suffer from chemoresistance. Several studies have reported that Fn14 could regulate migration, invasion, and angiogenesis in cancer cells. However, its functional role in chemoresistance of HGSOC is still unknown.

**Methods:**

The expression of Fn14 in tissue specimens was detected by IHC. CCK-8 assay was performed to determine changes in cell viability. Apoptosis was measured by flow cytometry. The potential molecular mechanism of Fn14-regulated cisplatin resistance in HGSOC was investigated using qRT-PCR, western blotting, and Co-IP assays. The role of Fn14 in HGSOC was also investigated in a xenograft mouse model.

**Results:**

In this study, we found that Fn14 was significantly downregulated in patients with cisplatin resistance. Patients with low Fn14 expression were associated with shorter progression-free survival and overall survival. Overexpression of Fn14 suppressed cisplatin resistance in OVCAR-3 cells, whereas knockdown of Fn14 did not affect cisplatin resistance in SKOV-3 cells. Interestingly, Fn14 could exert anti-chemoresistance effect only in OVCAR-3 cells harboring a p53-R248Q mutation, but not in SKOV-3 cells with a p53-null mutation. We isolated and identified primary cells from two patients with the p53-R248Q mutation from HGSOC patients and the anti-chemoresistance effect of Fn14 was observed in both primary cells. Mechanistic studies demonstrated that overexpression of Fn14 could reduce the formation of a Mdm2-p53-R248Q-Hsp90 complex by downregulating Hsp90 expression, indicating that degradation of p53-R248Q was accelerated via Mdm2-mediated ubiquitin-proteasomal pathway.

**Conclusion:**

Our findings demonstrate for the first time that Fn14 overcomes cisplatin resistance through modulation of the degradation of p53-R248Q and restoration of Fn14 expression might be a novel strategy for the treatment of HGSOC.

**Electronic supplementary material:**

The online version of this article (10.1186/s13046-019-1171-6) contains supplementary material, which is available to authorized users.

## Background

Ovarian cancer is the most lethal of all gynecological malignancies and the fifth most common cause of tumor-related death among women worldwide [1]. High-grade serous ovarian cancer (HGSOC) accounts for nearly 80% of all ovarian cancers, mostly diagnosed at advanced stages with poor prognosis [2]. Compared to other subtypes, HGSOC is more aggressive with shorter progression-free survival [3]. Despite surgical debulking and administration of platinum-based chemotherapy, majority of the patients suffer from drug resistance and disseminated disease leading to their death in less than 5 years. In addition, efforts aimed at developing new therapeutic approaches have largely been unsuccessful. As a representative of platinum anticancer drugs, cisplatin plays a crucial role in the treatment of HGSOC in clinical chemotherapy. Therefore, an improved understanding of the molecular mechanisms underlying cisplatin-resistance in HGSOC has the potential to significantly affect patient outcomes.

Fibroblast growth factor-inducible 14 (Fn14, also known as TNFRSF12A), receptor for cytokine tumor necrosis factor-like weak inducer of apoptosis (TWEAK), is a member of the tumor necrosis factor receptor super-family [4, 5]. Fn14 expression has been widely detected in different mammalian tissues and most prominently in immunocytes, liver, kidney and heart. Several studies have found that Fn14 is related to inflammation and autoimmune disease by regulating pro-inflammatory cytokine secretion [6, 7]. Moreover, Fn14 is expressed in most solid tumors and reports have identified that Fn14 could regulate migration, invasion and angiogenesis in cancer cells [8, 9]. These studies mainly focused on the function of Fn14 in tumor metastasis and little is known about its role in chemoresistance. Cisplatin acts by forming a platinum complex inside a cell which binds to DNA. When DNA is cross-linked in this manner, the cell undergoes systemic cell death via apoptosis. The dysregulation of apoptotic pathways could result in post-target resistance to cisplatin [10]. Furthermore, Fn14 could effectively inhibit tumor growth by promoting apoptosis in hepatocellular carcinoma and endometrial carcinoma [11, 12], but little is known about the underlying molecular mechanisms. Based on these findings, we hypothesized that Fn14 might overcome cisplatin resistance in HGSOC by modulating cisplatin-induced apoptosis. In this study, we identified that Fn14 attenuates chemoresistance via enhancing cisplatin-induced apoptosis in HGSOC. Furthermore, we have uncovered a mechanism wherein Fn14 causes apoptosis by inducing the ubiquitylation and degradation of p53-R248Q. Our findings indicate that Fn14 might act as a therapeutic target to improve the efficacy of cisplatin resistance and prognosis in HGSOC patients with p53-R248Q mutation.

## Materials and methods

### Tissue specimens

Seventy-one paraffin-embedded tissue samples were collected from patients who underwent ovary debulking surgery and received systemic treatment with a cisplatin plus paclitaxel-based regimen at the Affiliated Renji Hospital of Shanghai Jiaotong University, Shanghai, China between January 2013 and December 2018. Histological characterization of all these 71 samples indicated that they were high-grade serous ovarian cancer samples. The criterion to classify these samples as cisplatin resistant or sensitive was based on the literature [13]. All specimens were re-evaluated independently by two experienced pathologists. Signed informed consent was obtained from all the patients involved in this study, and the experimental protocols were approved by the ethical committee of Renji Hospital.

### Cell lines and clinical samples

Human HGSOC cell lines were purchased from the Cell Bank of the Chinese Academy of Sciences (Shanghai, China). Tumor tissues from HGSOC patients were dissociated to single cells by enzymatic digestion. According to a previously described procedure [14], patient-1 and patient-2 cell lines were established from primary cells derived from patient-1 and patient-2 tumor samples, respectively. Cells were cultured in DMEM (Hyclone, GE Healthcare, UT, USA) supplemented with 10% fetal bovine serum (FBS) (HyClone) and penicillin/streptomycin antibiotic solution (1:100, Sigma Aldrich, St. Louis, MO, USA) and incubated at 37 °C in a humidified atmosphere under 5% CO_2_ conditions.

### Immunohistochemistry (IHC)

IHC staining was performed on 4-μm sections of paraffin-embedded HGSOC samples to determine Fn14 expression level by using an anti-Fn14 antibody (Abcam, Cambridge, UK). In brief, the sections were subjected to standard procedures [15]. The sections were incubated with an anti-Fn14 antibody (1:100). PBS staining served as the negative control. Two pathologists conducted the IHC scoring procedures independently, in duplicate. Score criterion of IHC was performed as follows, the percentage of staining: 0, < 5%; 1, 5–25%; 2, 25–50%; 3, 51–65%; and 4, > 65%. The intensity of staining: 0 = negative staining, 1 = weak staining, 2 = moderate staining, and 3 = strong staining. The final score was determined by multiplying the scores of percentage of staining with the intensity of staining. Low expression was defined as a score between 0 to 4, whereas high expression was defined as a score between 5 to 12.

### CCK-8 assay

Eight thousand cells per well were seeded in a 96-well plate before cisplatin treatment. During detection, each well was replaced with 100 μL of fresh medium containing 10 μL of CCK-8 and incubated for 1 h. The absorbance was measured at 450 nm by a microplate reader from Thermo Scientific (Massachusetts, USA).

### Flow cytometry

For analysis of cell apoptosis, an Annexin V-FITC/PI apoptosis detection kit was used (BD Bioscience, San Jose, CA, USA). According to the manufacturer’s instructions, cells were collected and washed with binding buffer and then were incubated for 15 min with 5 μL of annexin V-FITC and 5 μL of PI. The apoptosis rate of the cells was examined by FAC Scan flow cytometry from Beckman Coulter (Brea, CA, USA).

### Quantitative real-time PCR

Total RNA was extracted from cells using Trizol (Takara, Japan). cDNA was synthesized using a PrimeScript RT Reagent Kit (Takara) according to the manufacturer’s instructions. Quantification of mRNA was performed using SYBR Premix Ex Taq II (Takara) and CFX96TM PCR detection system (Bio-Rad, Hercules, CA, USA). *GAPDH* served as a reference gene. Relative expression was calculated using the comparative ΔΔCT method. The following primers were used: p53F: 5′ TGAGCGCTTCGAGATGTTCC 3′, p53R: 5′ GACTGGCCCTTCTTGGTCTT 3′, MDR1F: 5′ ATATCAGCAGCCCACATCAT 3′, MDR1R: 5′ GAAGCACTGGGATGTCCGGT 3′, BAXF 5′ TCCACCAAGAAGCTGAGCGAG 3′, BAXR: 5′ GTCCAGCCCATGATGGTTCT 3′.

### Western blot analysis

RIPA buffer was used to lyse the cells and protein concentration of the cell lysate was measured by BCA protein assay kit (Bio-Rad Laboratories, Hercules, CA, USA). Protein extract (20–30 μg) was loaded on SDS-PAGE gels (10% or 12%) and the separated proteins were transferred onto a PVDF membrane. The membrane was blocked with 5% non-fat milk for 1 h. Antibodies were diluted as follows: anti-Fn14 (1:1000, no.4403; Cell Signaling Technology, Beverly, MA, USA), anti-Bcl-2 (1:1000, no.2872; Cell Signaling Technology), anti-Caspase-3 (1:1000, no.9662; Cell Signaling Technology), anti-MDM2 (1:1000, no.86934; Cell Signaling Technology), anti-Hsp70 (1:1000, no. 4872; Cell Signaling Technology), anti-Hsp90 (1:1000, no. 4874; Cell Signaling Technology), anti-ubiquitin (1:1000, no.3933; Cell Signaling Technology), anti-p53 (1:1000, no.sc-47,698; Santa Cruz, CA, USA), and GAPDH (1:1000, no. 2118; Cell Signaling Technology).

### Co-immunoprecipitation (co-IP) and ubiquitination assay

For Co-IP, 800 μg of protein extract was incubated overnight at 4 °C with primary antibody on a rotator. Antibodies were diluted as follows: anti-p53 (3 μg per 1000 μg protein, no.sc-47,698; Santa Cruz), MDM2 (1:100, no.86934; Cell Signaling Technology). Agarose beads (20 μL) were added to the above mixture and incubated for at least 2 h. The mixture was then centrifuged and washed three times with PBS. Samples were resuspended in 20 μL of gel loading buffer and western blotting was performed as indicated above.

### Sequencing of p53 in primary cells isolated from patient samples

Genomic DNA was extracted and purified according to the manufacturer’s instructions (Qiagen). The R248Q p53-coding region was sequenced using the primers as previously described [16].

### Transient transfection

Negative control siRNA and p53 siRNA were purchased from Integrated Biotech Solutions (Shanghai, China). Cells were transiently transfected using Lipofectamine 2000 (Invitrogen, Carlsbad, CA, USA) and were harvested after 48 h post-transfection. p53, Sense-1: 5′ GAGGGATGTTTGGGAGATGTA 3′, Sense-2: 5′ GAGGGATGTTTGGGAGAT-GTA 3′ Fn14, Sense-1: 5′ CAUCCAUUCUAGAGCCAGUCUTT 3′, Sense-2 5′ GAGGGAGA-AUUUAUUAAUAAATT 3′, Mdm2, Sense: 5′ AGGCAAAUGUGCAAUACCAUU 3′.

### Lentiviral infections

The control and overexpression Fn14 lentivirus were purchased from Gikai gene (Shanghai, China). The lentivirus was introduced into OVCAR-3 and primary cells by adding to cell growth medium. Then, stably overexpressing Fn14 cells or control cells were selected using medium containing 1 μg/ml puromycin (Sigma-Aldrich, Milwaukee, WI, USA).

### Animal experiment

Five weeks-old BALB/c nude mice were subcutaneously injected with 3 × 10^6^ OVCAR-3 cells. Once the tumors reached a volume of approximately 80–100 mm^3^ (14 days post-injection), the mice were randomly divided into two groups (*n* = 5) and administered chemotherapy. Cisplatin (4 mg/kg) was administered intraperitoneally twice a week up to 4 weeks. The tumor volume was calculated with the formula: V = (length × width^2^)/2.

### TUNEL assay

Sections from OVCAR-3 xenografts described above were processed using the In Situ TUNEL detection kit according to the manufacturer’s instructions (Beyotime, Shanghai, China). Positively stained cells were identified by bright fluorescence co-localized to a DAPI positive nucleus.

### Statistical analysis

The analyses were performed using Prism 7.0 software (GraphPad, Inc., San Diego, CA, USA). Unpaired Student’s t test was performed for comparison between two groups. Fisher’s exact test was performed for clinicopathological data analysis. Values of *p* < 0.05 were considered as statistically significant. Kaplan–Meier analysis was performed to evaluate the survival. Data are presented as mean ± SEM for three independent experiments.

## Results

### Loss of Fn14 coincided with chemoresistance and poor prognosis of HGSOC

**Fig. 1:**
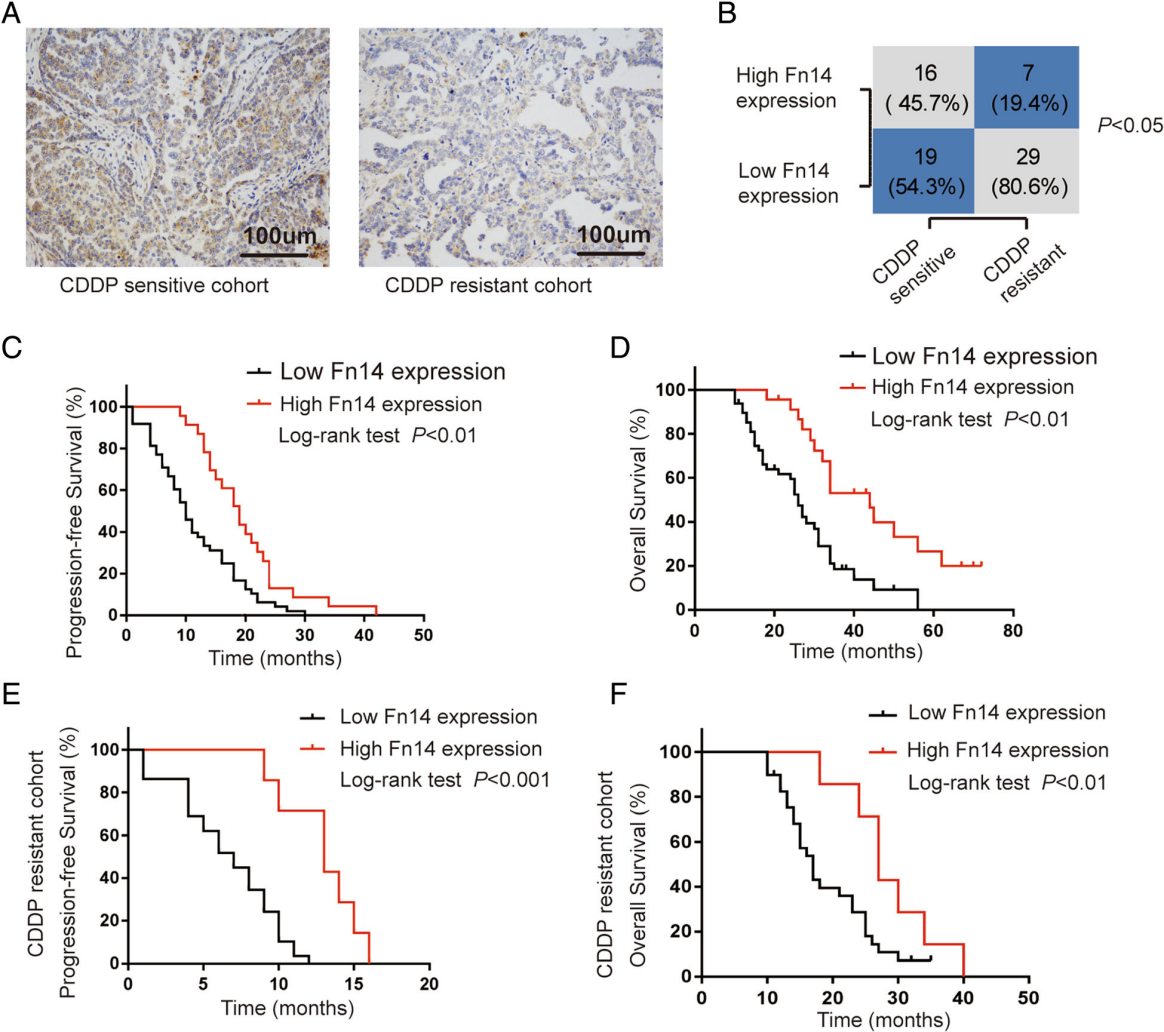
Loss of Fn14 coincided with chemoresistance and poor prognosis of HGSOC. (**a**) and (**b**) Distribution of Fn14 expression in cisplatin (CDDP) sensitive group and CDDP) resistant group, ^*^p < 0.05. (**c**) and (**d**) Survival outcome analysis showed that HGSOC patients with overexpression of Fn14 exhibited better progression-free survival and overall survival compared with those who had low expression of Fn14, ^**^p < 0.01. (**e**) and (**f**) Survival analysis showed that patients with overexpression of Fn14 exhibited better progression-free survival and overall survival compared with those who had low expression of Fn14 in CDDP resistant cohort, ^**^p < 0.01, ^***^p < 0.001, respectively. Fisher’s exact test was performed for clinicopathological data analysies. Values of *p* < 0.05 were considered as statistically significant. Kaplan–Meier analysis was used performed to evaluate the survival

To determine the function of Fn14 in cisplatin-resistant HGSOC, a panel of 71 HGSOC cases was studied by immunohistochemistry. We then analyzed the correlation between Fn14 expression and clinicopathological characteristics of HGSOC. As shown in Table 1, Fn14 expression in early-stage (I-II) patients was significantly higher than that in patients with advanced stage cancer (III-IV) (*p* < 0.01), whereas differences based on the patient age, ascites and residual lesions, were insignificant. Moreover, our analysis of high-grade serous ovarian tumors indicated that patients with cisplatin resistance exhibited low levels of Fn14 protein (IHC analysis) (*p* < 0.05, Fig. 1a and b). The Kaplan–Meier analysis showed that low Fn14 expression is significantly associated with poor progression-free survival (*p* < 0.01) and overall survival (*p* < 0.01) (Fig. 1c and d). Consistently, we evaluated whether Fn14 expression contributed to patient survival in cisplatin-resistant cohort. Further, survival analysis revealed that high expression of Fn14 contributes to improved outcomes for patients with chemoresistance (Fig. 1e and f). Taken together, we have identified that Fn14 might regulate resistance against cisplatin in high-grade serous ovarian cancer.

**Table 1:** Clinicopathological features of HGSOC tissues with regard to the relative expression of Fn14

Patients’ characteristics	Total	Fn14 IHC (*n*)		*P*
	*n*	low expression	high expression	
	71	(*n* = 48, 67.6%)	(*n* = 23, 43.3%)	
Age(y)				
≤ 50	19	10 (52.6%)	9 (47.4%)	0.152
>50	52	38 (73.1%)	14 (26.9%)	
FIGO stage				
I-II	8	1 (12.5%)	7 (87.5%)	0.001
III-IV	63	47 (74.6%)	16 (25.4%)	
Ascites				
positive	58	41 (70.7%)	17 (29.3%)	0.327
negative	13	7 (53.8%)	6 (46.2%)	
Residual lesions				
<2 cm	62	42 (67.7%)	20 (32.3%)	> 0.999
≥ 2 cm	9	6 (66.7%)	3 (33.3%)

### The effects of Fn14 on cisplatin-resistance in HGSOC cells

**Fig. 2:**
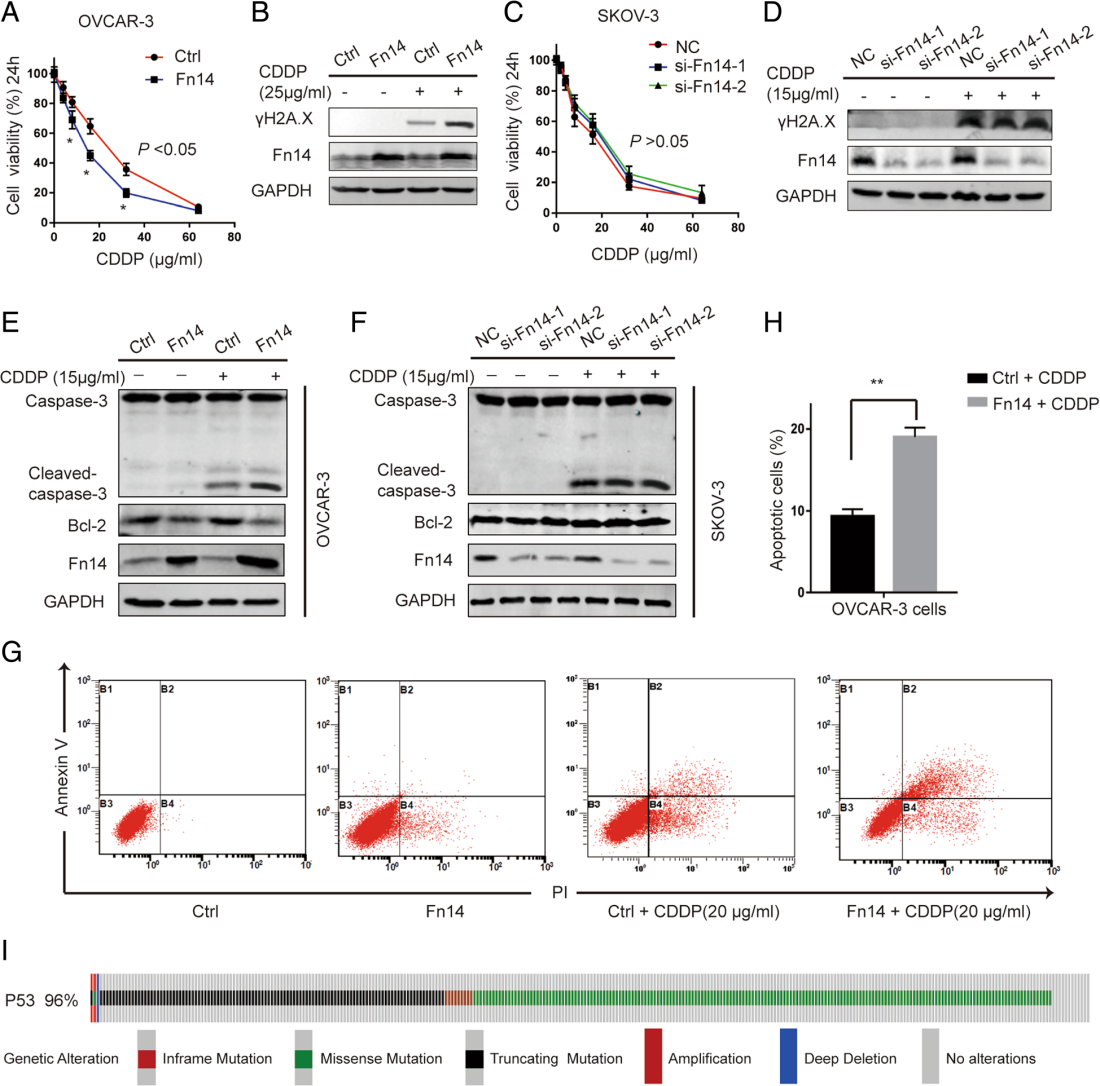
The effects of Fn14 on cisplatin-resistance in HGSOC cells. (**a**) CCK-8 assay was performed to detect cell viability in OVCAR-3 cells,^*^p < 0.05. (**b**) Western blot analysis detecting the DNA damage maker in OVCAR-3 cells infected with Fn14 lentivirus or the control lentivirus. (**c**) CCK-8 assay was performed to detect cell viability in SKOV-3 cells. (**d**) Western blot analysis detecting the DNA damage maker in SKOV-3 cells transfected with the Fn14 siRNA or negative control. (**e**) and (**f**) Western blot analysis detecting the activation of Caspase-3 and the expression of Bcl-2 in OVCAR-3 and SKOV-3 cells after diffident treatment. (**g**) and (**h**) Effects of Fn14 on apoptosis of OVCAR-3 cells were determined by flow cytometry analysis, ^**^p < 0.01. (**i**) Genetic Alteration of p53 in HGSOC. Unpaired Student’s t test was performed for comparisons between two groups

We examined Fn14 expression in a panel of human HGSOC cell lines, HEY, OVISE, OVCAR-3, and SKOV-3. SKOV-3 cells exhibited the highest endogenous expression of Fn14 and OVCAR-3 cells showed lowest expression of Fn14 (data not shown). Among these cell lines, OVCAR-3 is an appropriate model system to study drug resistance in ovarian cancer as indicated by American Type Culture Collection (ATCC). Considering aforementioned results, we chose SKOV-3 and OVCAR-3 cells for subsequent experiments. To determine whether Fn14 could sufficiently alleviate resistance to cisplatin in HGSOC cells, Fn14 was stably overexpressed in OVCAR-3 cells and was knocked down in SKOV-3 cells. We found that Fn14 overexpression significantly attenuated cell viability and markedly enhanced cisplatin-induced DNA damage measured by γH2AX in OVCAR-3 cells (Fig. 2a-b and Additional file 1: Figure S1A). Furthermore, Fn14 overexpression significantly increased cell apoptosis in response to cisplatin treatment (Fig. 2 G and H). In addition, the expression of caspase-3 and its cleaved form, a key mediator of apoptosis, was detected by western blotting. We observed that there was a significant increase in the expression of cleaved caspase-3 and decrease in the expression of anti-apoptotic protein Bcl-2 in OVCAR-3 cells (Fig. 2 E and Additional file 1: Figure S1C). To further confirm if Fn14 inhibits cisplatin resistance in ovarian cancer consistently, the results of knockdown of Fn14 were analyzed in SKOV-3 cells. Interestingly, down-regulation of Fn14 had little, if any, effect on cell viability and DNA damage as well as cisplatin-induced apoptosis in SKOV-3 cells (Fig. 2c, d, f and Additional file 1: Figure S1B, D).

### Fn14 inhibits cisplatin resistance in HGSOC primary cancer cells with p53-R248Q mutation

**Fig. 3:**
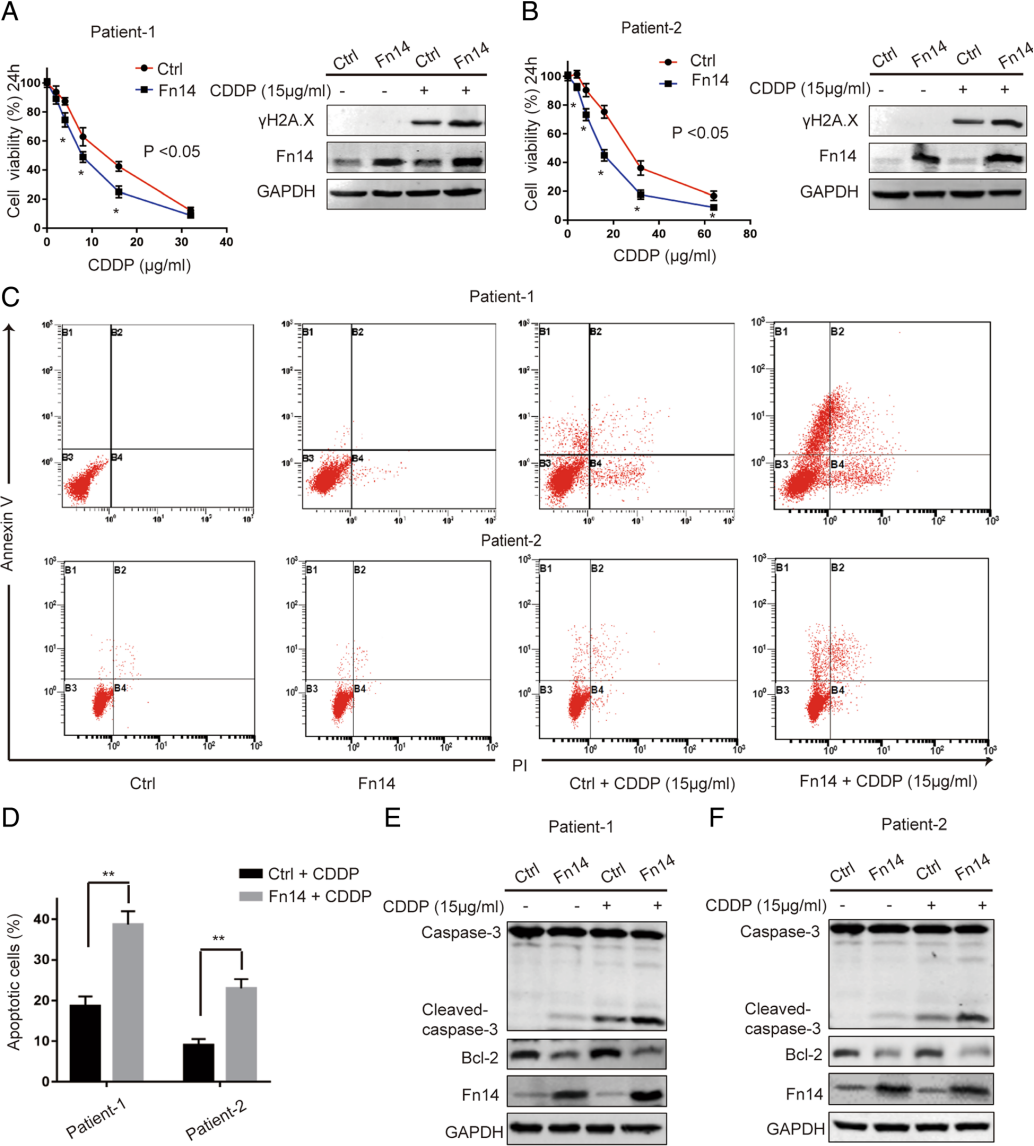
Fn14 inhibits cisplatin resistance in HGSOC primary cancer cells with p53-R248Q. (**a**) and (**b**) CCK-8 assay was performed to detect cell viability in primary cells with p53 R248Q after infection with Fn14 lentivirus or control, ^*^p < 0.05. Western blot analysis detecting the DNA damage maker in primary cells infected with Fn14 lentivirus. (**c**) and (**d**) Effects of Fn14 on apoptosis of primary cells were determined by flow cytometry analysis, ^**^p < 0.01. (**e**) and (**f**) Western blot analysis detecting the activation of Caspase-3 and the expression of Bcl-2 in primary cells after infection with Fn14 lentivirus. Unpaired Student’s t test was performed for comparisons between two groups

Next, we tried to investigate as to why Fn14 had a significant effect on cisplatin resistance in OVCAR-3 cells but not in SKOV-3 cells. Cisplatin is a DNA damaging agent dependent on p53 for potent activity and HGSOC presents with the highest prevalence of p53 mutations, where mutations are reported in more than 96% of the cases [17] (Fig. 2i). Therefore, we hypothesized that p53 mutations might contribute to the differential effects of Fn14 on cisplatin-resistance in HGSOC. We examined p53 mutation status in ovarian cancer cells using an online database (http://p53.iarc.fr/CellLines.aspx) (Additional file 7: Table S1). We identified that OVCAR-3 cells harbor p53-R248Q mutation and SKOV-3 cells harbor p53 null mutation. In addition, p53-R248Q mutation not only abolishes the tumor suppressive function, but also promotes cancer by acquiring gain-of-function (GOF) activities [18]. Compared to other frequent p53 hotspot mutations (R175, G245, G249, R273), patients with p53-R248Q mutation exhibited shorter progression free survival and overall survival in HGSOC [19]. Based on these findings, we examined whether Fn14 might alleviate cisplatin-resistance in HGSOC with p53-R248Q mutation. Due to limited access to HGSOC cells as well as clinical samples with p53-R248Q mutation, we isolated primary cells from a cohort of HGSOC patients (*n* = 18, Additional file 8: Table S2) bearing various p53 mutations and screened two primary cell colonies harboring p53-R248Q mutation, which were named patient-1 and patient-2 cells (Additional file 2: Figure S2B and C). By immunofluorescence staining analysis, it was confirmed that these primary cells were positive for cytokeratin-7 (CK-7), an epithelial marker and were negative for vimentin, a mesenchymal marker (Additional file 2: Figure S2A). We examined Fn14 expression and half maximal inhibitory concentration (IC_50_) of cisplatin in patient-1 and patient-2 cells. Both the primary cells weakly expressed Fn14 and were resistant to cisplatin (Additional file 2: Figure S2D and E). To further validate whether Fn14 could attenuate cisplatin-resistance in primary cells with p53-R248Q mutation, we established stable cell lines constitutively overexpressing Fn14 (Patient-1-Fn14 and Patient-2-Fn14) by using lentivirus. Upon cisplatin treatment, Fn14 overexpression significantly promoted cell death and DNA damage in both patient cell lines (Fig. 3a-b and Additional file 3: Figure S3A-B). Furthermore, Fn14 overexpression also significantly increased cell apoptosis and promoted the expression of cleaved caspase-3 after exposure to cisplatin in HGSOC primary cells (Fig. 3c-f and Additional file 3: Fig. S3C-D). These data suggested that Fn14 could alleviate cisplatin-resistance of HGSOC with p53-R248Q mutation.

### Fn14 attenuates cisplatin-resistance by down-regulation of p53-R248Q

**Fig. 4:**
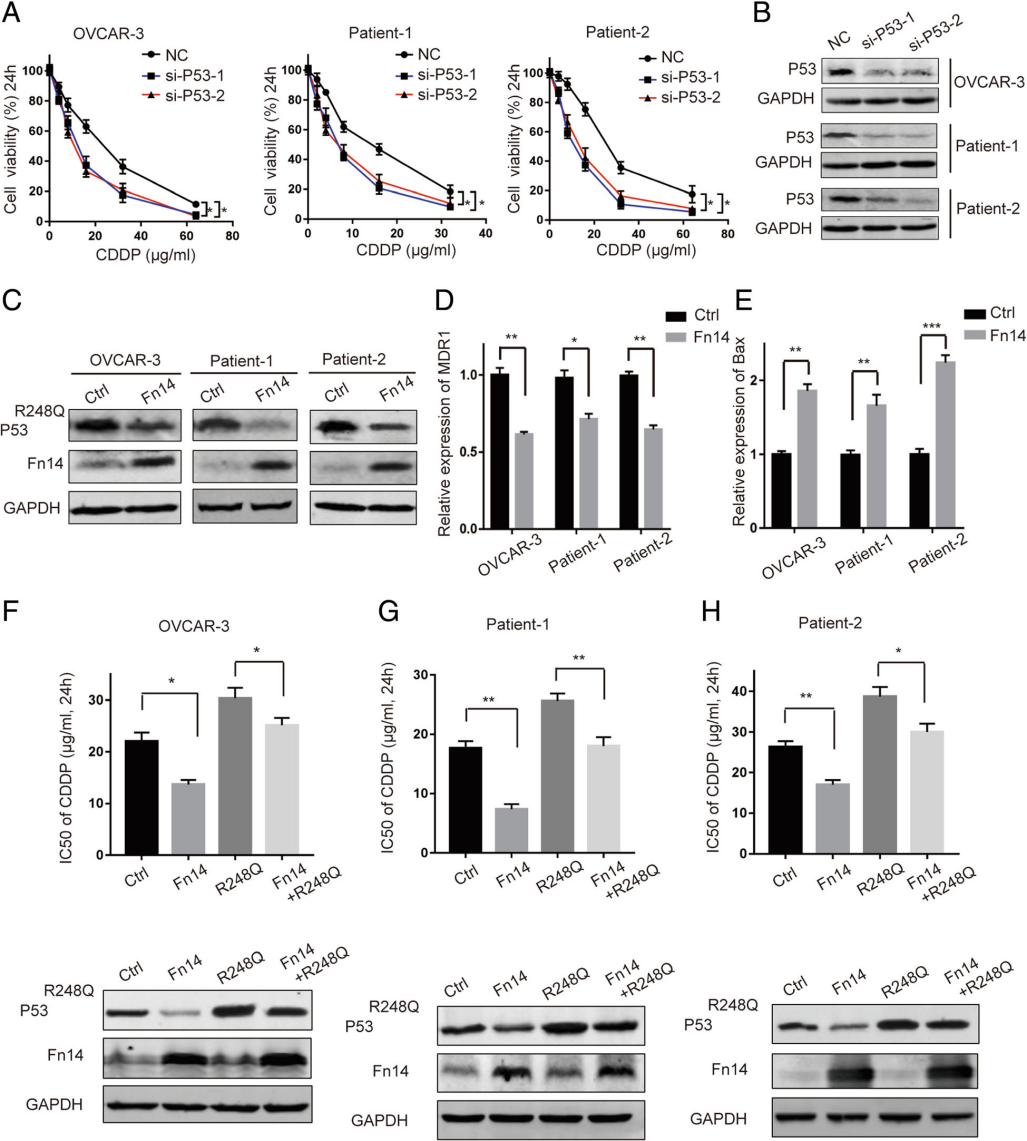
Fn14 inhibits cisplatin resistance in HGSOC primary cancer cells with p53-R248Q. (**a**) CCK-8 assay was performed to detect cell viability in HGSOC cells transfected with p53 siRNA or negative control. (**b**) Western blot showing the levels of p53 R248Q in HGSOC cells transfected with p53 siRNA. (**c**) Western blot analysis detecting the the expression of p53-R248Q in HGSOC cells after infection with Fn14 lentivirus. (**d**) and (**e**) qRT-PCR detecting the expression of p53-R248Q target genes in HGSOC cells after infection with Fn14 lentivirus, ^*^p < 0.05, ^**^p < 0.01, ^***^p < 0.001. (**f**-**h**) CCK-8 assay was performed to detect IC50 of CDDP in HGSOC cells co-infected with Fn14 and p53 R248Q lentivirus, ^*^p < 0.05, ^**^p < 0.01. Unpaired Student’s t test was performed for comparisons between two groups

Owing to mutp53 GOF, mechanisms of mutp53-induced chemoresistance include enhanced drug efflux and metabolism, inhibiting apoptosis, promoting survival, up-regulating DNA repair and elevating microenvironmental resistance [20]. When we knocked down the expression of p53-R248Q in HGSOC cells, significantly less viable cells were observed than the negative control in response to cisplatin treatment (Fig. 4a-b and Additional file 4: Figure S4A). To determine whether p53-R248Q is regulated by Fn14, we overexpressed Fn14 in OVCAR-3 cells and observed that the expression of p53-R248Q was less pronounced than the control cells (Fig. 4c and Additional file 4: Figure S4B). Similar results were obtained in patient-1 and patient-2 cells (Fig. 4c and Additional file 4: Figure S4B). Furthermore, we examined the expression of p53-R248Q target genes related to chemoresistance following Fn14 perturbation. Reduced expression of p53-R248Q protein caused due to Fn14 overexpression corresponded to repression of p53-R248Q transcriptional targets [20], MDR1 and Bax (Fig. 4d-e). To ascertain whether the effect of Fn14 on cisplatin resistance is mediated through p53-R248Q dependent mechanism, we found that increased p53-R248Q expression could rescue the effect of Fn14 overexpression on cisplatin resistance in HGSOC cells (Fig. 4f-h and Additional file 4: Figure S4C). Therefore, Fn14 overexpression in HGSOC cells could attenuate cisplatin-resistance by targeting p53-R248Q.

### Fn14 enhances ubiquitylation and degradation of p53-R248Q by down-regulation of heat-shock protein (Hsp) 90

**Fig. 5:**
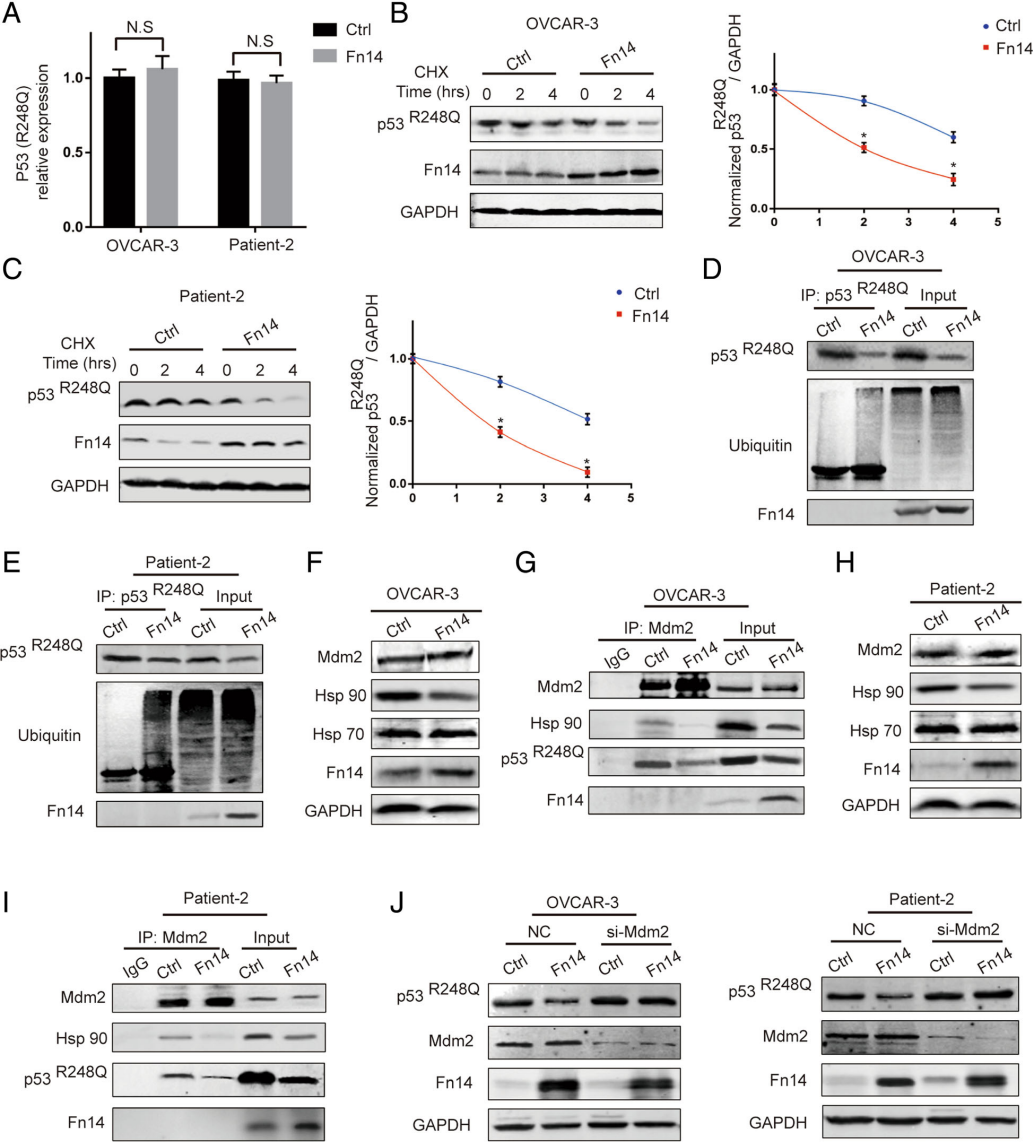
Fn14 enhances ubiquitylation and degradation of p53-R248Q by down-regulation of Hsp90. (**a**) qRT-PCR detecting the mRNA expression of p53-R248Q in HGSOC cells after infection with Fn14 lentivirus (**b**) and (**c**) Upon CHX treatment, western blot analysis detecting the expression of p53-R248Q in HGSOC cells after infection with Fn14 lentivirus. (**d**) and (**e**) Co-IP analysis detecting the level of ubiquitination p53 R248Q in HGSOC cells infected with Fn14 lentivirus. (**f**) and (**h**) western blot analysis detecting the expression of Hsp90 in HGSOC cells after infection with Fn14 lentivirus. (**g**) and (**i**) Co-IP analysis detecting the expression of mutp53-Mdm2-Hsp90 complex in HGSOC cells infected with Fn14 lentivirus. Unpaired Student’s t test was performed for comparisons between two groups. (**j**) Western blot analysis detecting the expression of p53-R248Q in HGSOC cells after transfection with Mdm2 siRNA

Next, we explored the molecular mechanisms by which Fn14 regulated the expression of p53-R248Q protein. Down-regulation of p53-R248Q due to Fn14 overexpression was not a result of transcriptional repression (Fig. 5a). From these results, we hypothesized that Fn14 might down-regulate p53-R248Q expression at the post-translational level. Upon cycloheximide (CHX) blockade of de novo protein synthesis, Fn14 overexpression accelerated the degradation of p53-R248Q resulting in decreased half-life of p53-R248Q protein in OVCAR-3 cells (Fig. 5b). Similar results were obtained in patient-2 cells (Fig. 5c). Given the fact that mutant p53 often escapes from Mdm2- mediated ubiquitin-proteasomal degradation [18], we further hypothesized that Fn14 might promote the ubiquitination-dependent degradation of p53-R248Q protein. As shown in Fig. 5d, the amount of ubiquitin that coimmunoprecipitated with p53-R248Q was significantly increased in OVCAR-3 cells with Fn14 overexpression. Consistently, overexpression of Fn14 enhanced p53-R248Q-ubiquitin association in patient-2 cells (Fig. 5e). Hsp70 and Hsp90 could stably interact with mutant p53 and form complexes, suggesting that formation of Hsp-p53-R248Q-Mdm2 complex leads to escape from Mdm2-mediated degradation of tumor cells. To explore the mechanism by which Fn14 promoted ubiquitination-dependent degradation of p53-R248Q protein, the expression levels of Hsp70, Hsp90 and Mdm2 were examined. We found that HGSOC cells overexpressing Fn14 have reduced levels of Hsp90 protein (Fig. F, H and Additional file 5: Figure S5A). We next found that Fn14 overexpression significantly reduced the formation of Hsp90-p53-R248Q-Mdm2 complex thereby promoting ubiquitin-mediated degradation of p53-R248Q in OVCAR-3, patient-2 and SKOV-3 cells (Fig. 5g, i and Additional file 5: Figure S5C). In addition, to ascertain whether the effect of Fn14 overexpression on degradation of p53-R248Q was mediated through Mdm2 dependent mechanism, we silenced Mdm2 expression and found that it could abolish the effect of Fn14 overexpression on degradation of p53-R248Q in HGSOC cells (Fig. 5j and Additional file 5: Figure S5B). Together, these data indicate that Fn14 enhances ubiquitylation and degradation of p53-R248Q by down-regulating Hsp90.

### Overexpression of Fn14 alleviates cisplatin resistance in vivo

**Fig. 6:**
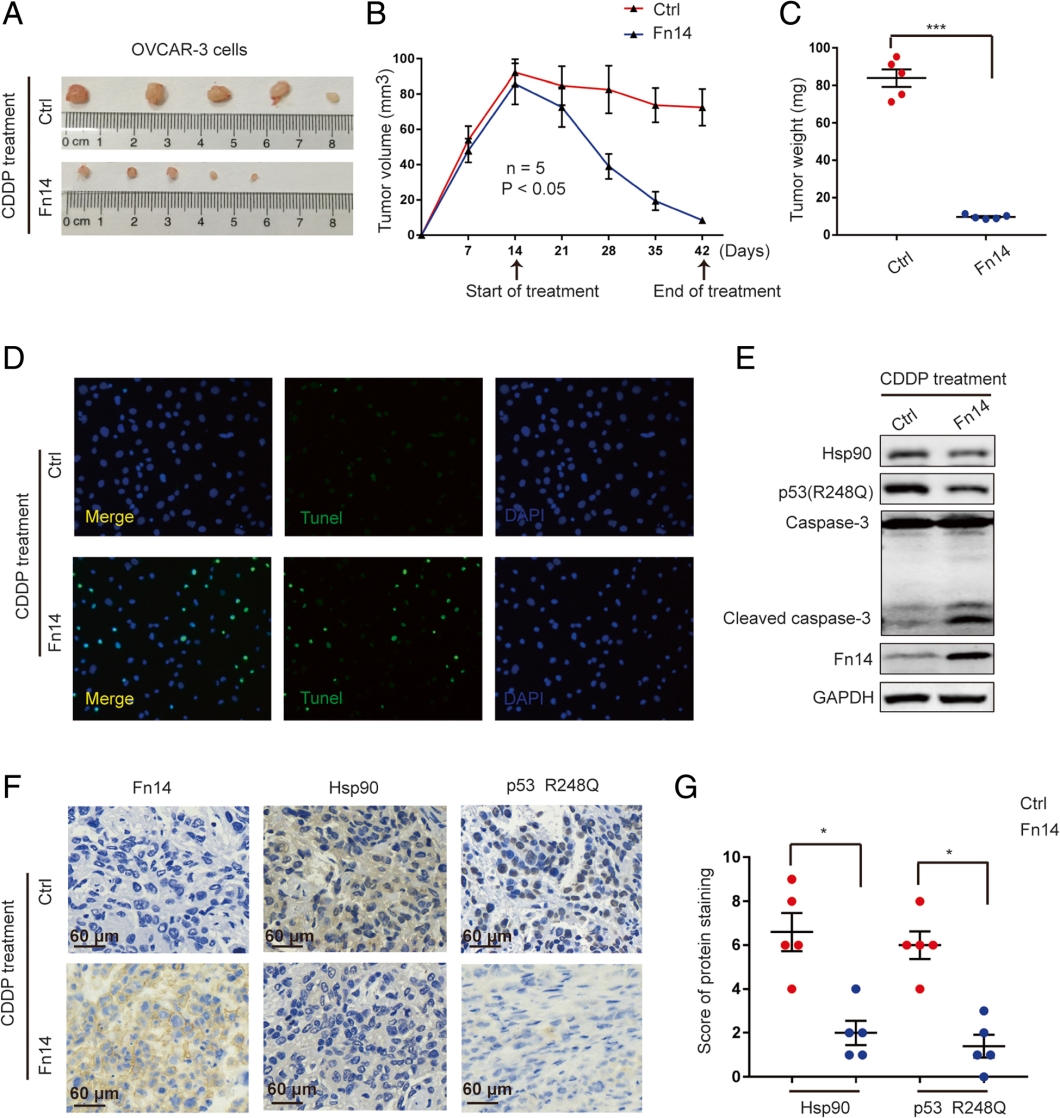
Overexpression Fn14 alleviates cisplatin resistance in vivo. (**a**-**c**) Macrograph, volume and weight of tumors in both two groups. (**d**) Tunel assay was used to determine the CDDP-induced DNA damage in the tumors of each group. (**e**) Western blot analysis detecting the activation of Caspase-3 and the expression of p53 R248Q and Hsp90 in tumor tissues in both two groups. (**f**) and (**g**) Tumors of each group were immunohistochemically tested for Fn14, Hsp90 and p53 R248Q, ^*^p < 0.05. Unpaired Student’s t test was performed for comparisons between two groups

Nude mice were injected with OVCAR-3 cells overexpressing Fn14. Upon cisplatin treatment, tumor volumes and tumor weight in subcutaneous tumors were significantly decreased (Fig. 6a-c). DNA fragmentation indicative of apoptosis, was detected by TUNEL assay in tumor tissues. TUNEL-positive cells were significantly higher in the Fn14 overexpression group exposed to cisplatin (Fig. 6d). Consistently, overexpression of Fn14 in OVCAR-3 tumor tissues resulted in elevated levels of cleaved caspase 3 in response to cisplatin treatment (Fig. 6e and Additional file 6: Figure S6A). In addition, IHC data showed that Fn14 overexpression could more effectively inhibit the expression of Hsp90 and p53-R248Q in tumor tissues than that in control tissues (Fig. 6f, g and Additional file 6: Figure S6B). Taken together, these data suggest that targeting Fn14 could alleviate cisplatin resistance in mutant p53-R248Q HGSOC cancer cells.

## Discussion

In HGSOC, patients often fail to respond to available chemotherapeutic agents and suffer from recurrence in 2 years after initial anticancer treatment. Therefore, identifying the molecular mechanism of chemoresistance to improve the prognosis of patients is critical. In this study, we found that Fn14 was down-regulated in HGSOC patients with cisplatin resistance. Functional studies revealed that ectopic overexpression of Fn14 alleviated cisplatin resistance in HGSOC cells with p53-R248Q mutation both in vitro and in vivo. Mechanistically, we found that Fn14 could reduce p53-R248Q protein expression by enhancing ubiquitin-mediated degradation of p53-R248Q by down-regulation of Hsp90. Thus, our study identified that Fn14 played an important role in abolishing cisplatin resistance in HGSOC and also uncovered the mechanisms of regulation of mutant p53 degradation.

Remarkably, studies have shown that Fn14 plays a crucial role in various malignant tumors. Fn14 contributes to regulating human tumor cell migration, invasion, and metastasis. However, its role in chemoresistance has not yet been studied in HGSOC. Cisplatin-induced DNA damage leads to the activation of a multibranched signaling cascade with proapoptotic outcomes and anti-apoptosis is one of the important mechanisms of chemoresistance leading to therapeutic failure. Fn14 appears to exhibit tumor suppressive activity by inducing apoptosis in endometrial cancer, colon carcinoma and hepatocellular carcinoma [11, 12, 21], suggesting that Fn14 might affect chemoresistance. As expected, we found that patients with low expression of Fn14 in HGSOC are more likely to exhibit cisplatin resistance, suggesting that Fn14 might be a biomarker for cisplatin resistance. Moreover, in our study (*n* = 71), patients with high Fn14 expression had significantly longer progression-free survival and overall survival. To clarify the suppressive mechanism of Fn14 in chemoresistance, overexpression of Fn14 in OVCAR-3 cells inhibited chemoresistance by increasing cisplatin-induced DNA damage and apoptosis. Unexpectedly, knockdown of Fn14 in SKOV-3 cells had no effect on chemoresistance. This discrepancy suggests that the suppressive role of Fn14 varies in different cancer types and other unreported mechanisms may be involved in the anti-chemoresistant effects of Fn14 in HGSOC. Cisplatin is a DNA damaging agent dependent on p53 for potent activity and HGSOC patients harbor p53 mutations. Of note, we found that Fn14 lost its anti-chemoresistance function in SKOV-3 cells harboring p53 null mutation. Moreover, overexpression of Fn14 markedly affected cisplatin resistance in OVCAR-3 cells bearing p53-R248Q mutation. Taken together, these data strongly demonstrate that Fn14 inhibits chemoresistance in HGSOC with p53-R248Q mutation. To further validate these findings, we isolated and identified primary cells with p53- R248Q mutation from HGSOC patient samples. Furthermore, overexpression of Fn14 in primary cells alleviated chemoresistance by promoting DNA damage and apoptosis. Therefore, these data indicate that Fn14 attenuated cisplatin resistance in HGSOC patients with p53-R248Q mutation.

P53 mutations can demonstrate abnormal GOF properties to facilitate oncogenesis and chemoresistance and this phenomenon has been widely confirmed by many in vivo and in vitro experiments [22]. Mutant p53 protein can misfold and form amyloid fibrillar aggregates in ovarian cancer and this aggregation promotes platinum resistance [23]. In addition, p53 V172F mutation also promotes cisplatin resistance in ovarian cancer [24]. In this study, knockdown of p53-R248Q significantly inhibited cisplatin resistance in OVCAR-3 cells and primary cells, which is consistent with previous studies elucidating the role of mutant p53. These findings indicate that Fn14 might affect cisplatin resistance by regulating p53-R248Q. Notably, in HGSOC cells, overexpression of Fn14 reduced the expression of p53-R248Q protein and its downstream target genes related to drug-resistance. Taken together, we demonstrated that Fn14 overcomes cisplatin resistance in HGSOC by down-regulating p53-R248Q.

Further, our study found that Fn14 decreased the expression of p53-R248Q protein, but had no effect on *p53* mRNA, indicating that Fn14 might regulate p53 expression at post-translational level. Wild-type p53 (wtp53) is regulated mainly by Mdm2, an E3 ubiquitin ligase that promotes the ubiquitylation-dependent proteasomal degradation [25]. Generally, wtp53 is short-lived and is rapidly degraded, but p53-R248Q is stable and accumulates increasingly in the nucleus by escaping MDM2-mediated degradation. The stabilization of p53-R248Q protein is a prerequisite for the manifestation of the gamut of its various gain-of-function (GOF) properties [18]. In our study, we found that Fn14 overexpression could accelerate degradation of p53-R248Q by increasing its ubiquitination. Several studies have revealed that wtp53 undergoes transient interactions with the HSPs. However, mutant p53 in tumors could stably interact with Hsp70 and Hsp90 forming p53-R248Q-Mdm2-Hsp complex to escape Mdm2-mediated degradation [26]. We identified that Fn14 overexpression could down-regulate the expression of Hsp90 in HGSOC cells. We thus speculated that Fn14 might disrupt the p53-R248Q-Mdm2-Hsp90 complex. Subsequent Co-IP experiments substantiated this hypothesis in OVCAR-3 and primary cells, suggesting that Fn14 overexpression could elevate Mdm2-mediated p53 ubiquitination and degradation by targeting Hsp90.

## Conclusions

Collectively, our findings successfully demonstrate for the first time that Fn14 could overcome cisplatin resistance through modulating the degradation of p53- R248Q and restoration of Fn14 expression might be a novel strategy for the treatment of HGSOC. Certainly, apart from the role of Fn14 revealed in our study, it has a multitude of other biological functions. Further studies are warranted to investigate the mechanism of down-regulation of Hsp90 by Fn14.

## Additional files

Additional file 1: **Figure S1.** The effects of Fn14 on cisplatin-resistance in HGSOC cells. (A)-(D) Statistical data of Western Blot. (TIF 769 kb)

Additional file 2: **Figure S2.** Characterization of primary HGSOC cells and identification of p53 status in primary cells. (A) Primary cells were photographed by light micrographs and CK7 (green) and Vimentin (red) expression was determined by Immunofluorescence. PI (blue) staining shows the nuclei, magnification of 200×. (B) and (C) The sequence of R248Q p53-coding region in primary cells. (D) Expression of Fn14 in four HGSOC cells was determined by western blot. (E) CCK-8 assay was performed to detect IC50 of CDDP in HGSOC cells. (TIF 2666 kb)

Additional file 3: **Figure S3.** Fn14 inhibits cisplatin resistance in HGSOC primary cancer cells with p53-R248Q. (A)-(D) Statistical data of Western Blot (TIF 743 kb)

Additional file 4: **Figure S4.** Fn14 inhibits cisplatin resistance in HGSOC primary cancer cells with p53-R248Q. (A)-(C) Statistical data of Western Blot. (TIF 815 kb)

Additional file 5: **Figure S5.** Fn14 could reduce the formation of Mdm2-p53-R248Q-Hsp90. (A)-(B) Statistical data of Western Blot. (C) Co-IP analysis detecting the expression of mutp53-Mdm2-Hsp90 complex in HGSOC cells infected with p53-R248Q lentivirus. (TIF 1031 kb)

Additional file 6: **Figure S6.** Overexpression Fn14 alleviates cisplatin resistance in vivo. (A) Statistical data of Western Blot (B) IHC images of tumors of each group (TIF 14600 kb)

Additional file 7: **Table S1.** P53 status in ovarian cancer cell lines. (TIF 16289 kb)

Additional file 8: **Table S2.** List of clinical samples used in this study. (TIF 16280 kb)

## Abbreviations

ATCC: American Type Culture Collection
CCK-8: Cell Counting Kit-8
CDDP: Cisplatin
CHX: Cycloheximide
CK-7: Cytokeratine-7
Fn14: Fibroblast growth factor-inducible 14
GOF: Gain-of-function
HGSOC: High grade serous ovarian cancer
HSP: Heat shock protein
IHC: Immunohistochemistry
TWEAK: Tumor necrosis factor-like weak inducer of apoptosis
Wtp53: Wild-type p53
