# Supplementary figures and images for: Fn14 overcomes cisplatin resistance of high-grade serous ovarian cancer by promoting Mdm2-mediated p53-R248Q ubiquitination and degradation
Source: J Exp Clin Cancer Res. 2019 Apr 25;38:176. doi: 10.1186/s13046-019-1171-6 (PMC6485139; doi:10.1186/s13046-019-1171-6)

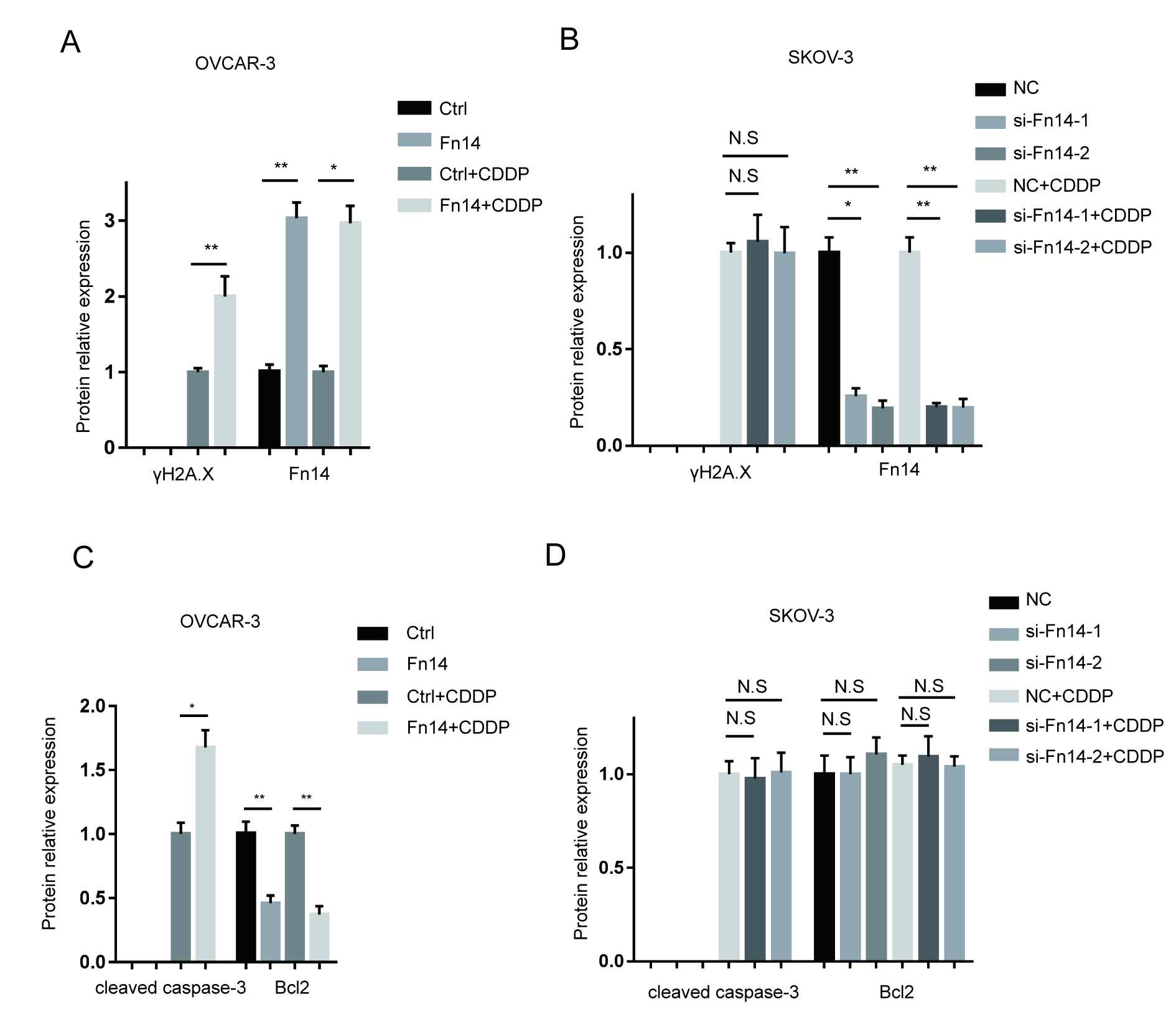

Supplement: Supplementary file 1 — Figure S1. The effects of Fn14 on cisplatin-resistance in HGSOC cells. (A)-(D) Statistical data of Western Blot. (TIF 769 kb) [file 13046_2019_1171_MOESM1_ESM.tif]

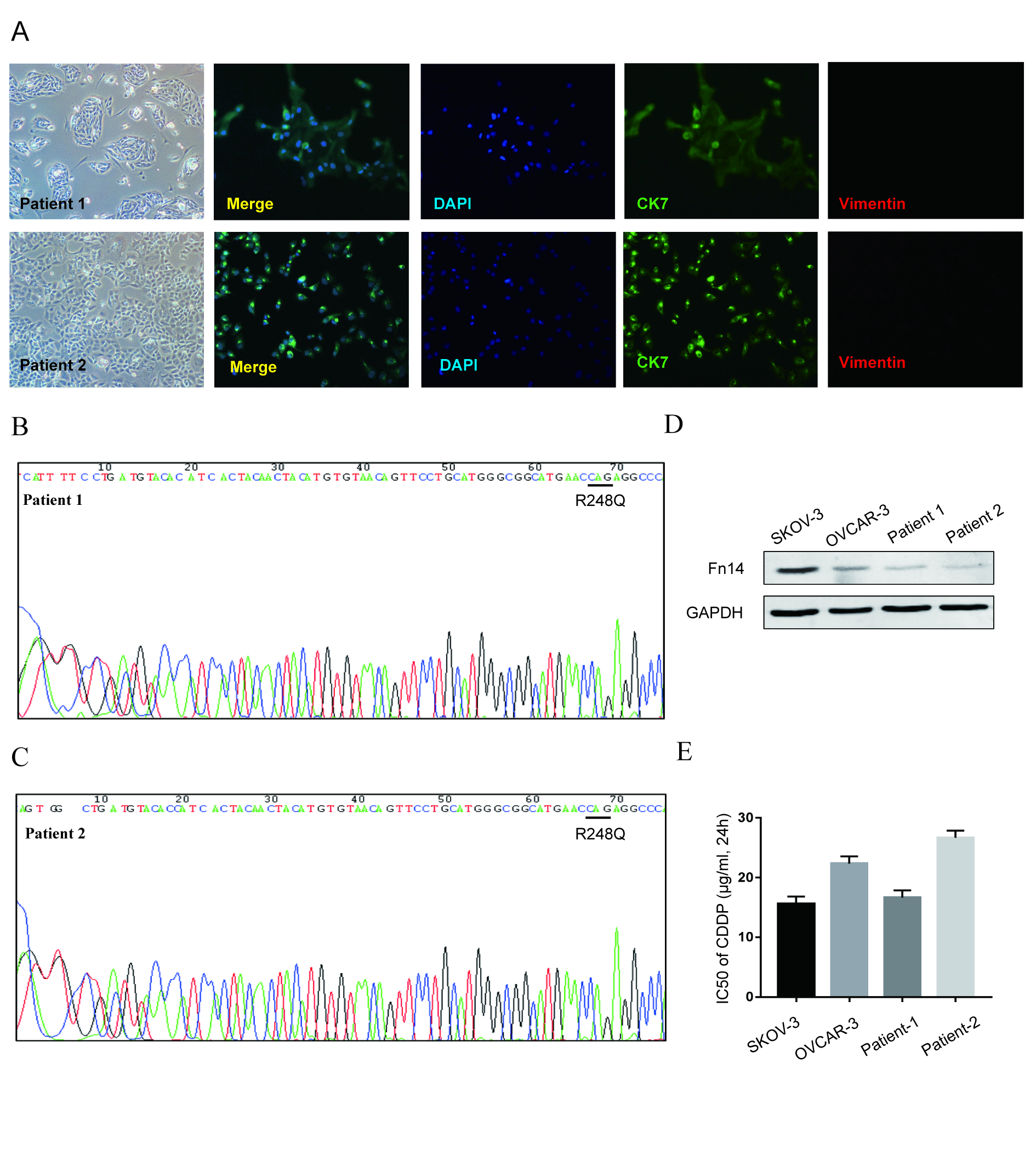

Supplement: Supplementary file 2 — Figure S2. Characterization of primary HGSOC cells and identification of p53 status in primary cells. (A) Primary cells were photographed by light micrographs and CK7 (green) and Vimentin (red) expression was determined by Immunofluorescence. PI (blue) staining shows the nuclei, magnification of 200×. (B) and (C) The sequence of R248Q p53-coding region in primary cells. (D) Expression of Fn14 in four HGSOC cells was determined by western blot. (E) CCK-8 assay was performed to detect IC50 of CDDP in HGSOC cells. (TIF 2666 kb) [file 13046_2019_1171_MOESM2_ESM.tif]

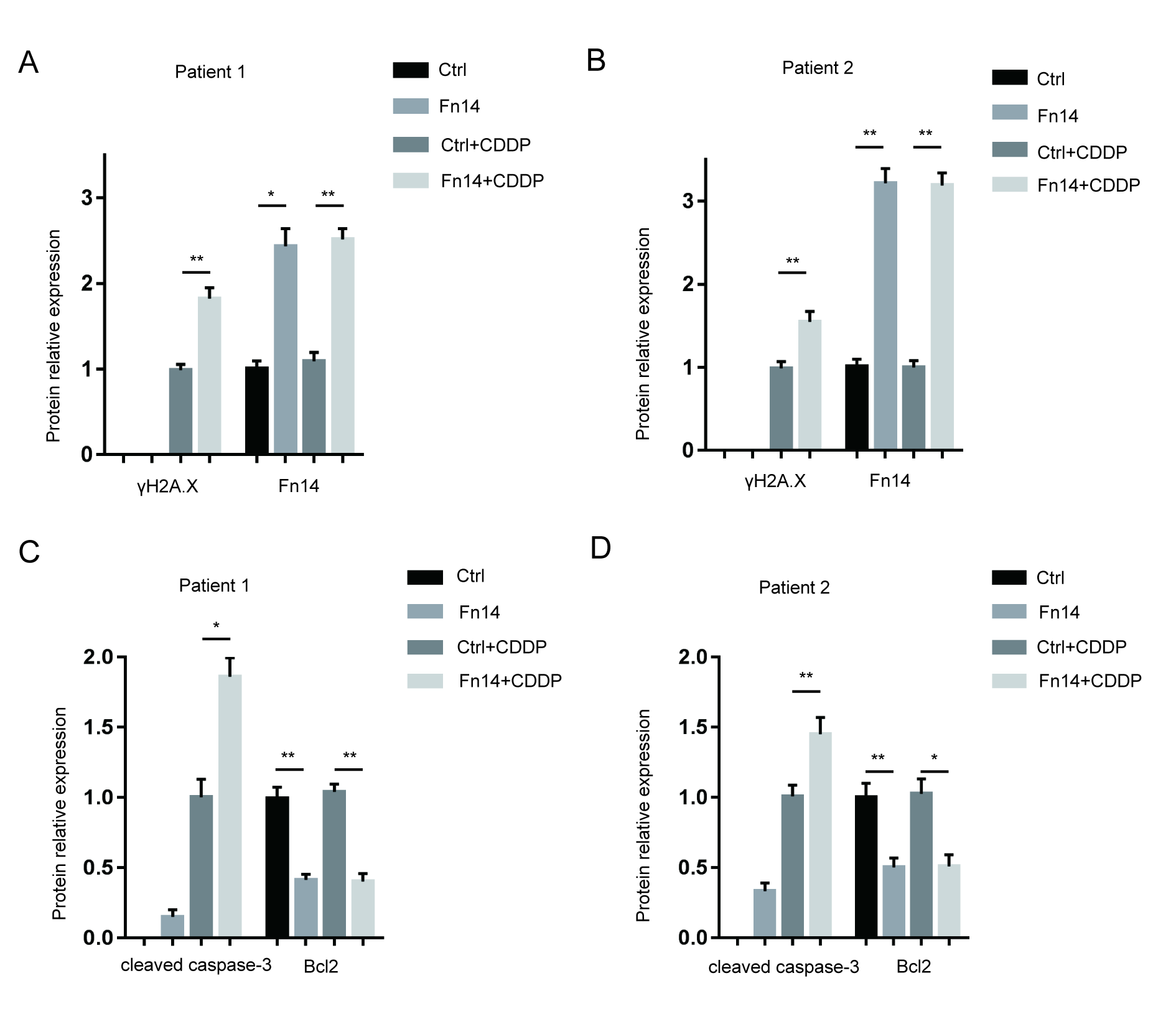

Supplement: Supplementary file 3 — Figure S3. Fn14 inhibits cisplatin resistance in HGSOC primary cancer cells with p53-R248Q. (A)-(D) Statistical data of Western Blot (TIF 743 kb) [file 13046_2019_1171_MOESM3_ESM.tif]

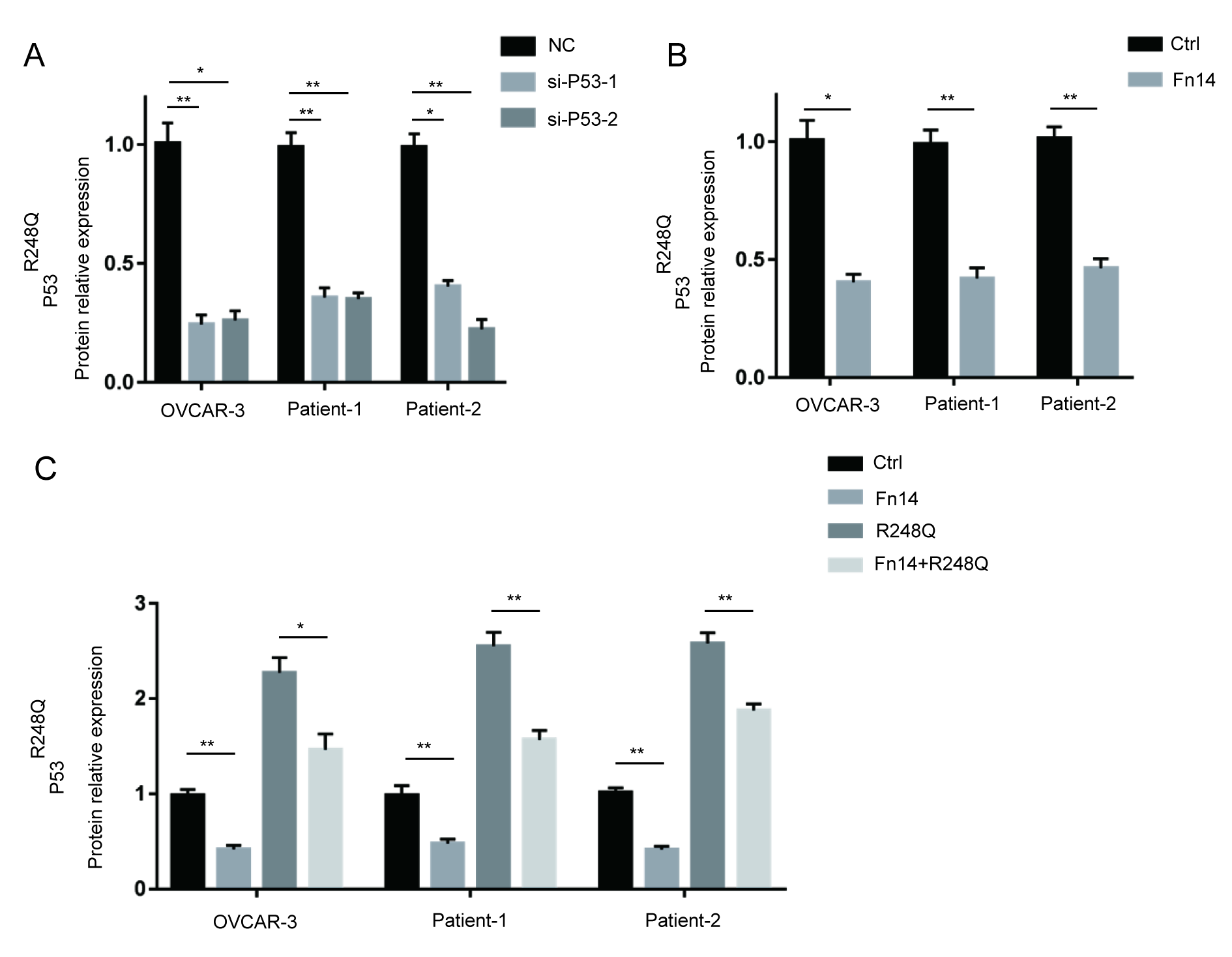

Supplement: Supplementary file 4 — Figure S4. Fn14 inhibits cisplatin resistance in HGSOC primary cancer cells with p53-R248Q. (A)-(C) Statistical data of Western Blot. (TIF 815 kb) [file 13046_2019_1171_MOESM4_ESM.tif]

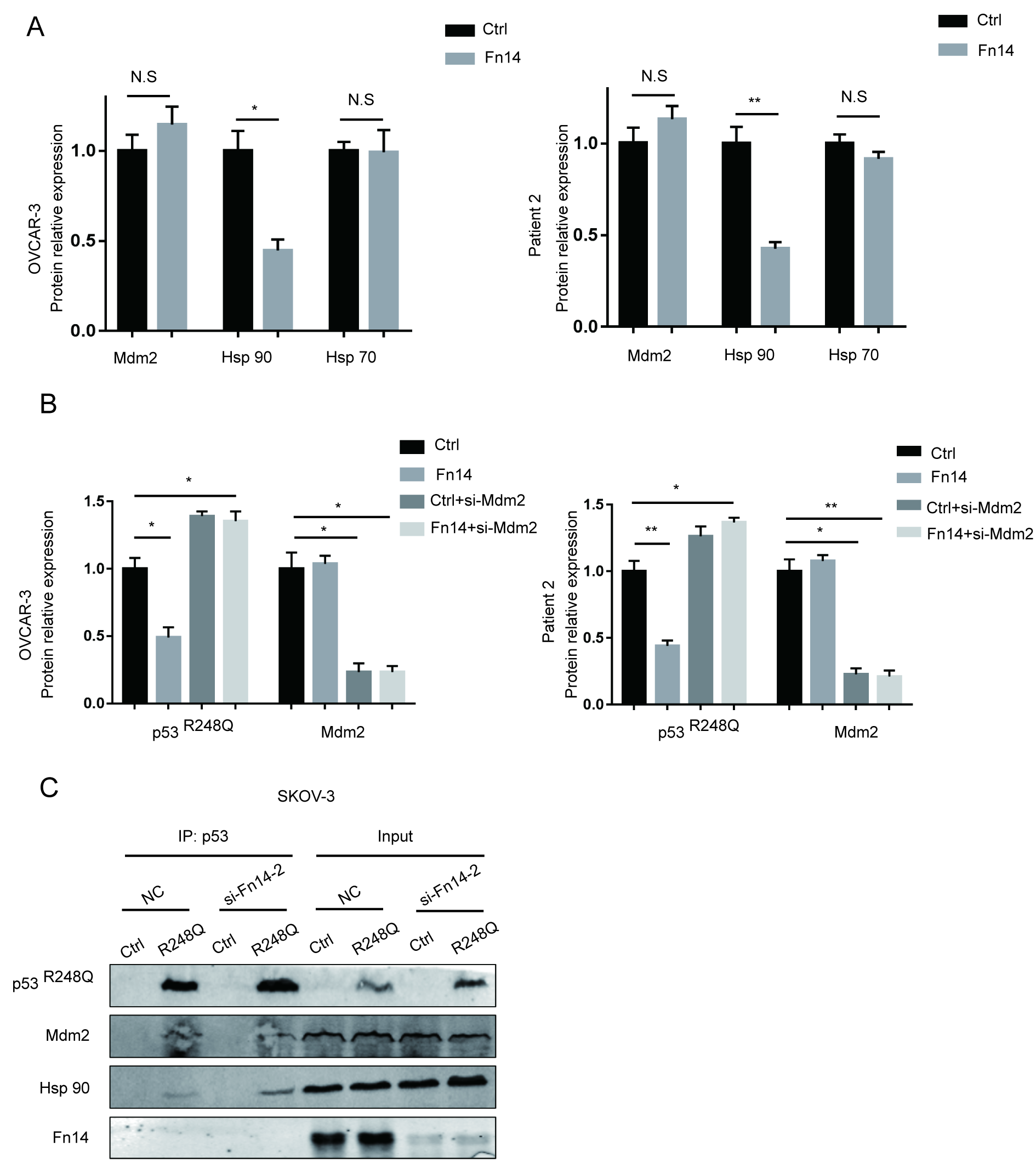

Supplement: Supplementary file 5 — Figure S5. Fn14 could reduce the formation of Mdm2-p53-R248Q-Hsp90. (A)-(B) Statistical data of Western Blot. (C) Co-IP analysis detecting the expression of mutp53-Mdm2-Hsp90 complex in HGSOC cells infected with p53-R248Q lentivirus. (TIF 1031 kb) [file 13046_2019_1171_MOESM5_ESM.tif]

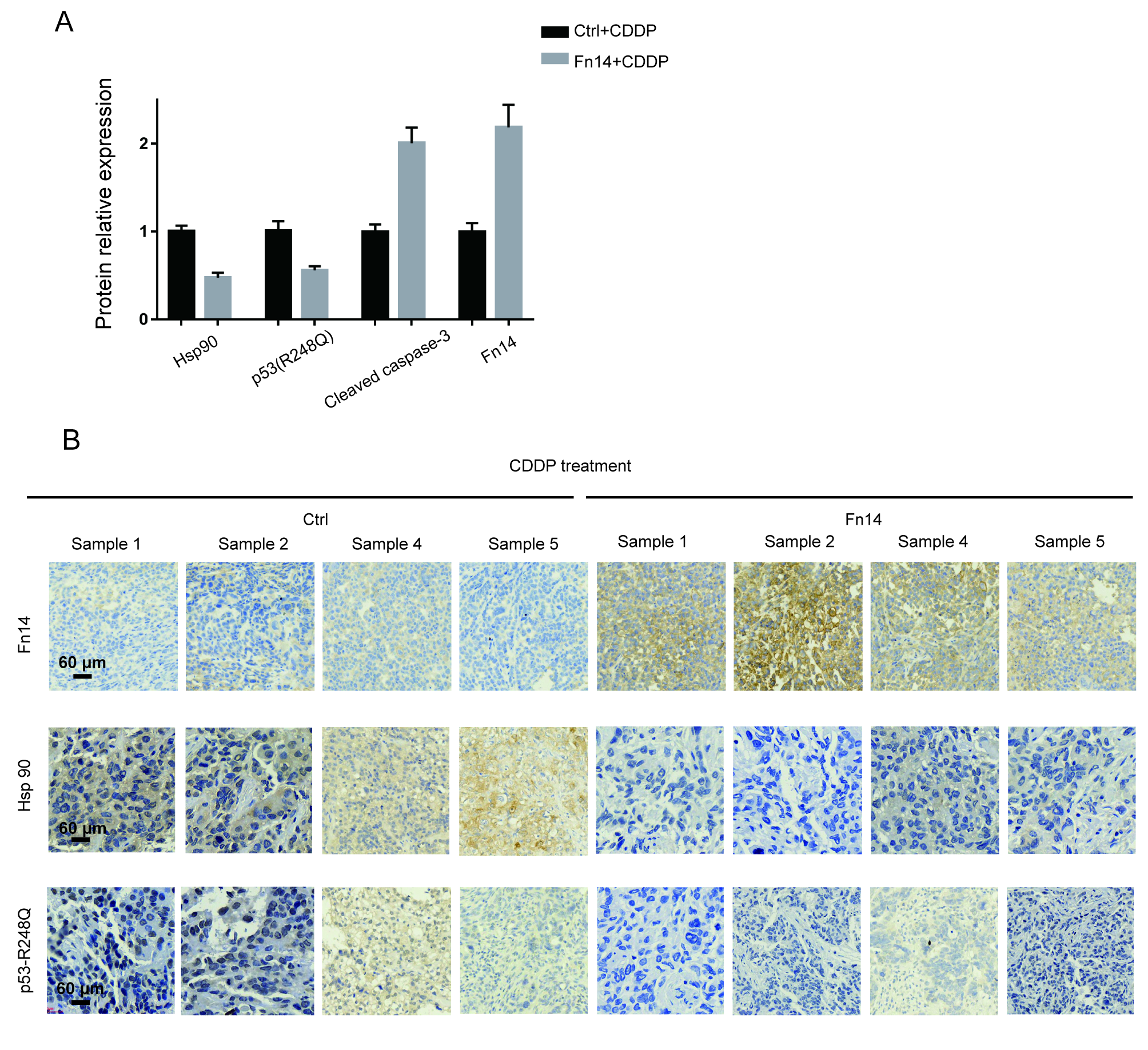

Supplement: Supplementary file 6 — Figure S6. Overexpression Fn14 alleviates cisplatin resistance in vivo. (A) Statistical data of Western Blot (B) IHC images of tumors of each group (TIF 14600 kb) [file 13046_2019_1171_MOESM6_ESM.tif]

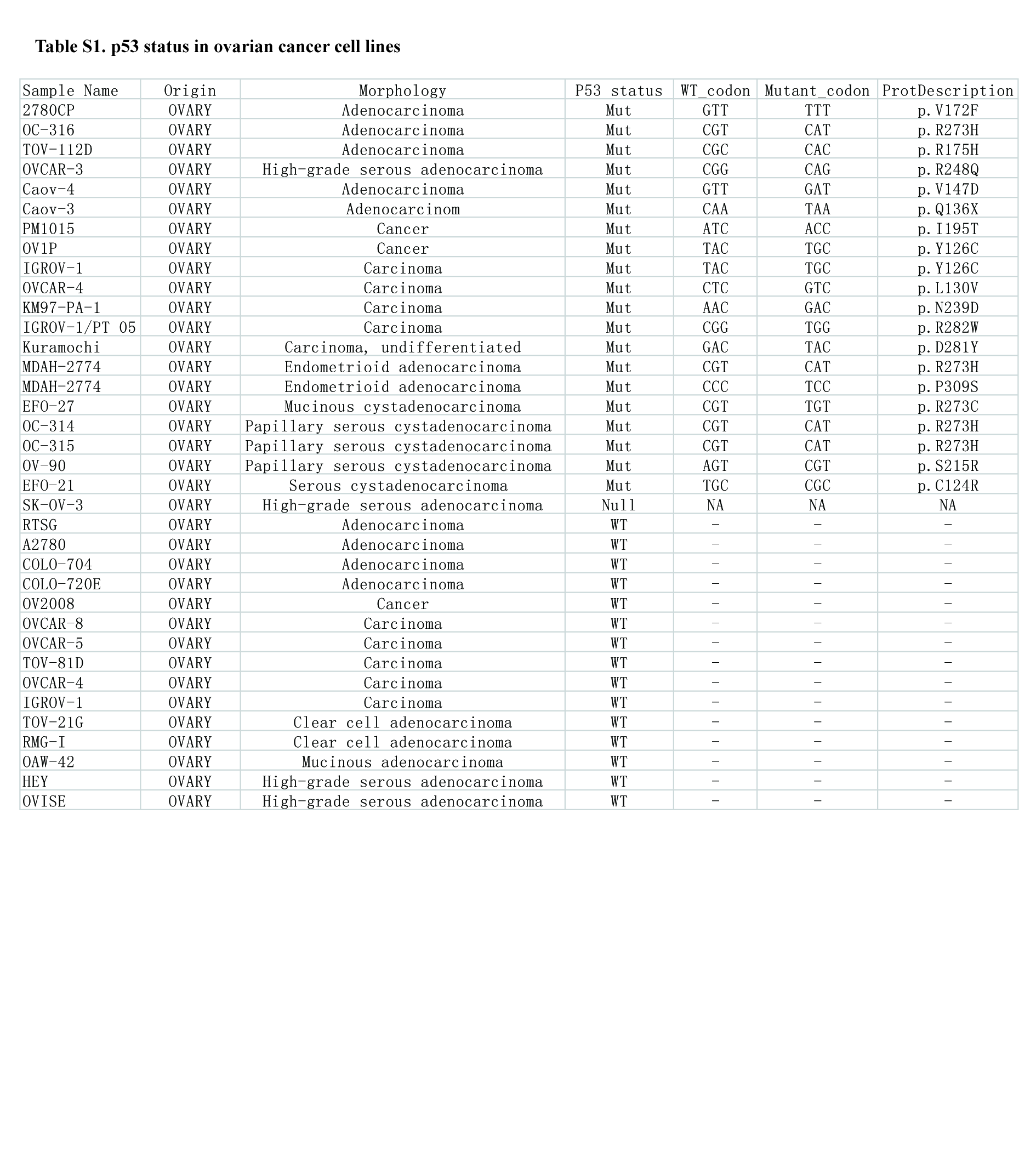

Supplement: Supplementary file 7 — Table S1. P53 status in ovarian cancer cell lines. (TIF 16289 kb) [file 13046_2019_1171_MOESM7_ESM.tif]

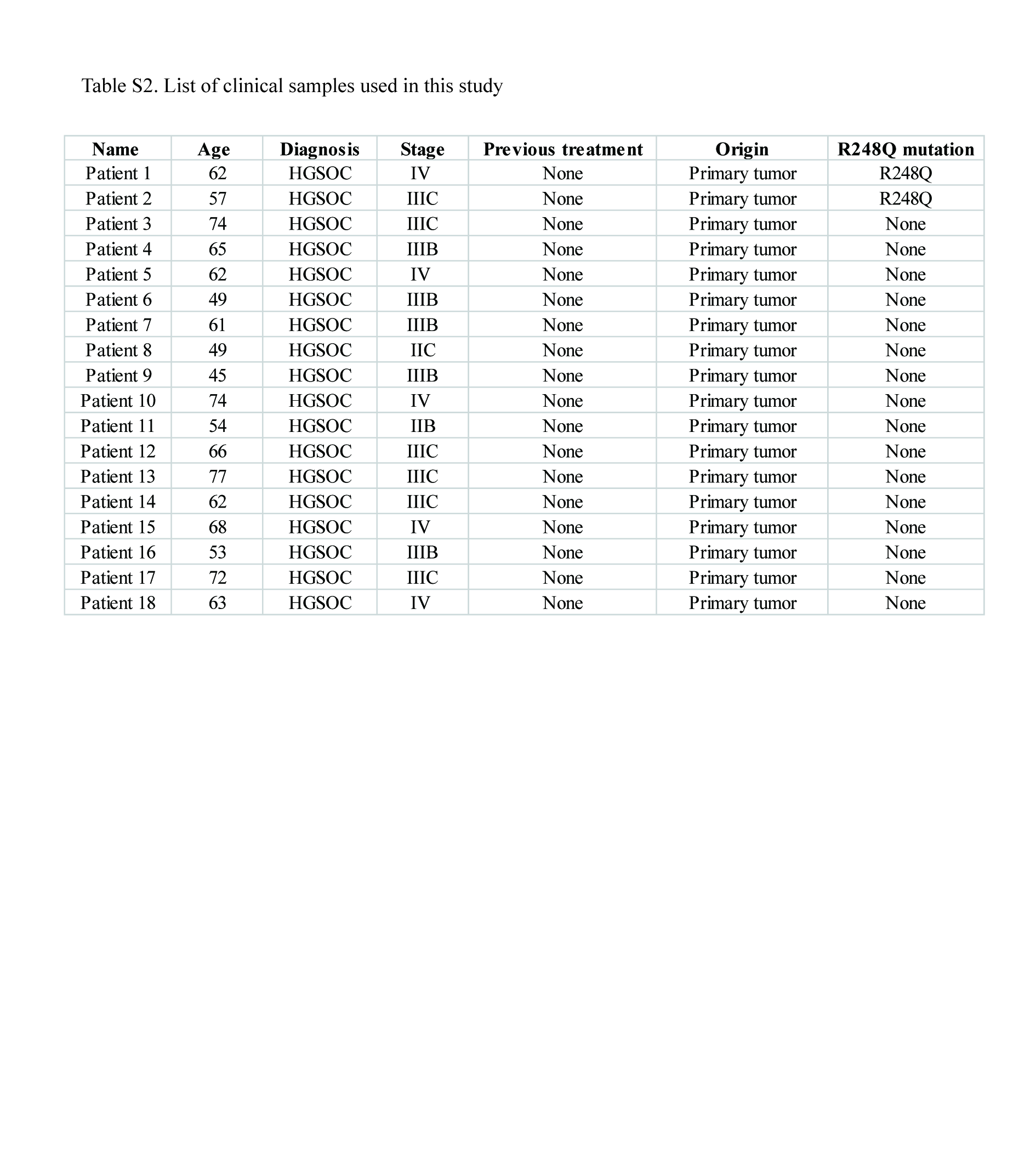

Supplement: Supplementary file 8 — Table S2. List of clinical samples used in this study. (TIF 16280 kb) [file 13046_2019_1171_MOESM8_ESM.tif]
